# Poor oral health may prolong COVID-19 illness

**DOI:** 10.1186/s12967-022-03310-0

**Published:** 2022-03-07

**Authors:** Shipra Gupta, Timo Sorsa, Ella Brandt, Ismo T. Räisänen, Ritin Mohindra, Kapil Goyal

**Affiliations:** 1 Unit of Periodontics, Oral Health Sciences Centre, Post Graduate Institute of Medical Education & Research, Chandigarh, India; 2 Department of Oral and Maxillofacial Diseases, University of Helsinki and Helsinki University Hospital, Helsinki, Finland; 3 Department of Internal Medicine, Post Graduate Institute of Medical Education & Research, Chandigarh, India; 4 Department of Virology, Post Graduate Institute of Medical Education & Research, Chandigarh, India

Letter to Editor,

We applaud the efforts of Wang et al. 2021 for their Mendelian randomization study on how periodontal disease increases the host susceptibility to COVID-19 along with its severity, published in Journal of Translational Medicine [1]. They report on the basis of genetic evidence, significant association of Periodontal disease with susceptibility to COVID-19 and its severity based on comparison of hospitalization versus population controls. We agree with the authors of the study that periodontal disease is a modifiable factor, and rigorous oral hygiene maintenance could go a long way in reducing the predisposition to COVID-19 related adverse complications.

Since the start of the pandemic in 2019, our team has been working diligently towards unravelling the intricacies of this association [2–6]. An association between periodontitis and higher risk of Intensive Care Unit admission, need for ventilation and death of COVID-19 patients, is now well documented [3]. Furthermore, data collected by us suggests that periodontitis may also prolong the number of days of COVID-19 illness (Fig. 1). Periodontally healthy patients had on average 4.15 days of illness (95% confidence interval: 3.18–5.12), patients with gingivitis 5.76 days (95% CI: 3.93–7.59) and patients with periodontitis 7.37 days (95% CI: 5.48–9.25). Thus, good oral health seems very much beneficial not only in the prevention of COVID-19 related adverse outcomes, but also in potentially shortening the duration of the disease. It could help reduce the economic burden on the individual and the society at large by cutting down on the number of days the patients stay away from work. Hence any diagnostic modality which could predictably screen the presence of periodontal disease could very well act as an alarm to encourage and motivate the patients to seek dental care before it has progressed to severe stage. Recently developed and validated active matrix metalloproteinase (aMMP)-8 point-of-care/chair-side biomarker technology provides possibilities for health care professionals in this regard [6]. These lateral flow immunoassay based kits are capable of diagnosing active periodontal disease at both full-mouth and site-specific levels and can aid in tailoring therapy as per patient requirements and at the same time sensitizing them towards the need to maintain oral health [6].

**Fig. 1:**
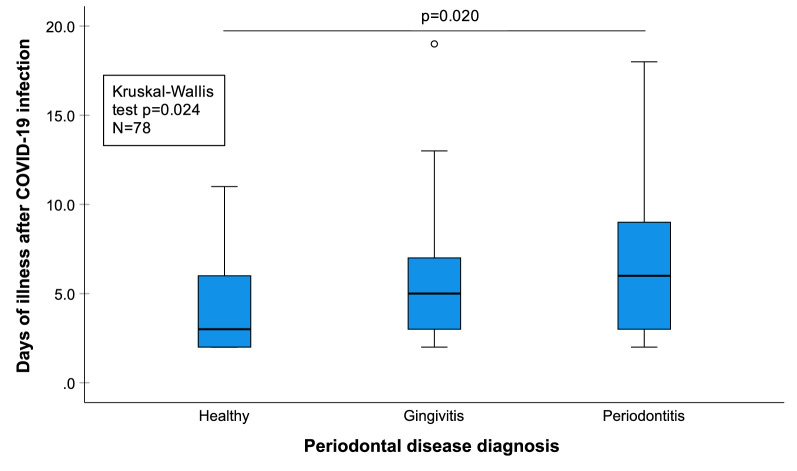
Periodontal disease diagnosis versus days of illness after COVID-19

These kind of preventive oral health strategies could aid in concentrating health care resources more efficiently on helping risk patients with additional comorbidities to have a better oral health and protection against infections caused by microbial pathogens, such as bacteria and viruses, and possibly reducing the risk of superinfections reported among severe COVID-19 cases. At the same time, it could also help to increase individuals’ knowledge of their possible periodontal disease and its current state and effects, which they often are unaware, and make it easier to motivate patients toward better oral hygiene and oral health. The selective identification and protection of these people at-risk individuals could be a cost-effective way of disease management, especially in areas with limited medical/dental facilities. Thus, this targeted prevention strategy could increase the general level of public health as well.

## Data Availability

Data and material can be achieved by email to the corresponding author (E-mail address: ismo.raisanen@helsinki.fi).
